# Beauvericin Immunotoxicity Prevention by *Gentiana lutea* L. Flower In Vitro

**DOI:** 10.3390/toxins15090538

**Published:** 2023-08-31

**Authors:** Giacomo Di Matteo, Alessandra Cimbalo, Lara Manyes, Luisa Mannina

**Affiliations:** 1 Food Chemistry Lab, Department of Chemistry and Technology of Drugs, Sapienza University of Rome, P. le Aldo Moro 5, 00185 Rome, Italy; giacomo.dimatteo@uniroma1.it (G.D.M.); luisa.mannina@uniroma1.it (L.M.); 2 Laboratory of Food Chemistry and Toxicology, Faculty of Pharmacy, Universitat de València, Avda Vicent Andrés Estellés s/n, 46100 Burjassot, Spain; alessandra.cimbalo@uv.es

**Keywords:** mycotoxin, *Gentiana lutea*, proteomics, LC-MS/MS-QTOF, dietary supplement

## Abstract

Beauvericin (BEA) is an emerging mycotoxin produced by some species of Fusarium genera that widely contaminates food and feed. *Gentiana lutea* is a protected medicinal plant known for its antioxidant and anti-inflammatory properties, which are attributed to its rich content of bioactive compounds. In order to evaluate the beneficial effects of *G. lutea* flower against BEA cytotoxicity, the aim of this study is to evaluate changes in protein expression after Jurkat cell exposure through a proteomics approach. To carry out the experiment, cells were exposed to intestinally digested *G. lutea* flower alone or in combination with the BEA standard (100 nM) over 7 days. Differentially expressed proteins were statistically evaluated (*p* < 0.05), revealing a total of 172 proteins with respect to the control in cells exposed to the BEA standard, 145 proteins for *G. lutea* alone, and 139 proteins when exposing the cells to the combined exposure. Bioinformatic analysis revealed processes implicated in mitochondria, ATP-related activity, and RNA binding. After careful analysis of differentially expressed proteins, it was evident that *G. lutea* attenuated, in most cases, the negative effects of BEA. Furthermore, it decreased the presence of major oncoproteins involved in the modulation of immune function.

## 1. Introduction

The European Food Safety Authority (EFSA) describes mycotoxins as toxic compounds produced by different types of fungi. They are food safety hazards that enter the food chain as a result of infection of harvests before or after collection and are typically found in foods such as cereals, dried fruits, nuts, and spices. A few have been legislated, but the vast majority have not [1]. In 2022, the EFSA launched a call for continuous collection of chemical contaminant occurrence data for food and feed, specifying some non-legislated mycotoxins, among them beauvericin (BEA) [2].

BEA is a cyclodepsipeptide mycotoxin, often found in high concentrations in raw and processed cereals, vegetables, fruits, and eggs because of fungal infection by Fusarium species [3]. In a review of total diet studies carried out in different countries focusing on mycotoxin determination, BEA was found in tomato products, nuts, and dried fruits in a concentration of approximately 2 μg/kg food [4]. In Europe, a study quantified BEA in a variety of ready-to-eat foods prepared with cereal products, vegetables, and meat [5]. Regarding its toxicity, BEA exposure in rodents induces immunotoxicity, reproductive disorders, and genotoxicity [6]. In order to reduce mycotoxin exposure damage in humans and animals, dietary antioxidant concomitant intake is a possible recommendation that has been explored recently [7,8].

In this sense, plants such as *Gentiana lutea* may be interesting candidates to be included as ingredients in foods vulnerable to mycotoxin contamination. *G. lutea*, known as “Great Yellow Gentian”, is a protected medical plant commonly found in mountainous regions of Central and Southern Europe and Western Asia. It grows on rich, relatively acidic soils on grassy alpine and subalpine continents. It shows antioxidant, antifungal, anti-inflammatory, stomachic, appetizer, and immunomodulatory properties. The plant can be used as a gallbladder and liver stimulant by affecting the efficiency of their function, as a remedy for digestive disorders, for mental and physical depression, and for exhaustion [9]. Šavikin et al. [10] discovered that extracts from leaves and flowers inhibited the growth of 15 out of 16 pathogenic microorganisms tested. They showed that the concentration of isogenistin was 10 times higher in flowers compared to leaves. Regarding the individual extract components, each tested compound did not possess a dominant role in the antimicrobial activity of raw extracts. Consequently, synergistic activity may be responsible for the inhibitory effect of the extracts.

Bioactive compounds are widely present at high concentrations in the flowers of *G. lutea*. A high concentration of phenolic acids was recorded in water extracts of flowers, with caffeic acid being the most abundant. Also, naringenin and hesperidin were both quantified as heteropolycyclic, aromatic bioflavonoids, antioxidants, and chemopreventive agents. The capacity of *G. lutea* for scavenging superoxide radicals is essentially due to the possibility of their occurrence through enzyme- or metal-catalyzed processes. Therefore, the flowers of *G. lutea* can be used in the food industry, as they are rich sources of natural preservatives and antimicrobial agents [11]. In fact, one important strategy to prevent mycotoxigenic fungal growth on food and feed is the addition of plant extracts rich in bioactive compounds with antifungal properties [12,13,14,15]. The aim of the present work was to evaluate the beneficial role of digested *Gentiana lutea* flower against BEA toxicity in a Jurkat lymphoblastoid cell line through a proteomic approach. For this purpose, cells were exposed to BEA (100 nM) alone or in combination with *G. lutea* (2%) for 7 days. More specifically, proteins were identified by using LC-MS/MS-QTOF, and the ones with different abundances with respect to the control were statistically filtered. Moreover, categorization of biological processes, molecular functions, and metabolic pathways where these proteins were involved was performed using the online Database for Gene and Protein Annotation, Visualization, and Integrated Discovery (DAVID).

## 2. Results

### 2.1. Differentially Expressed Proteins

**Figure 1:**
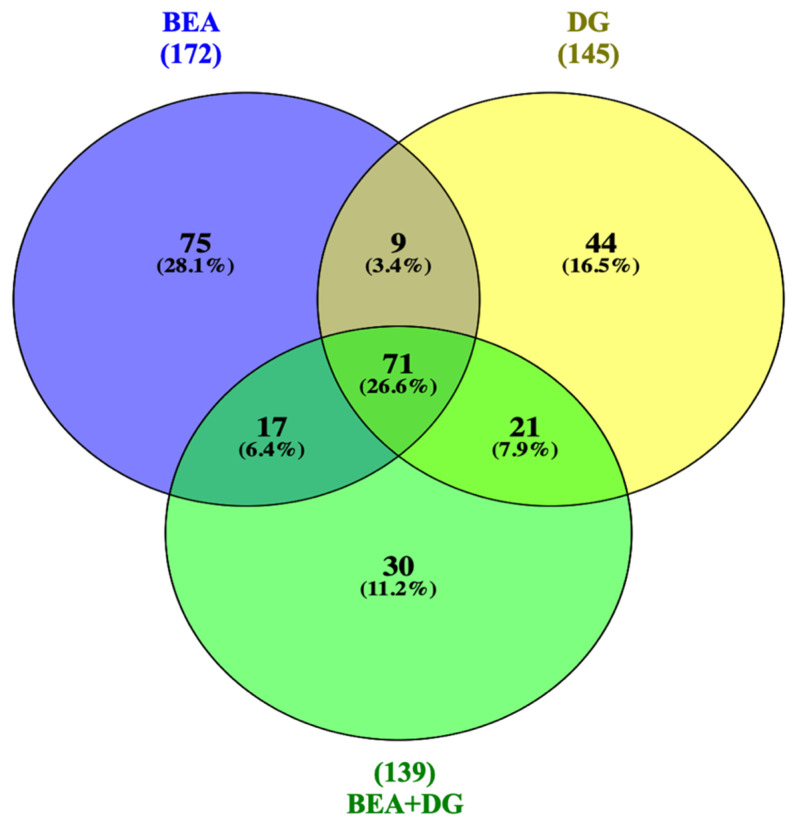
Venn diagram representation of DEPs for Jurkat cells exposed to BEA, DG, and BEA + DG compared to the control.

Differentially expressed proteins (DEPs) were obtained by comparing each exposure with the control group through unpaired t-test statistical analysis (*p* < 0.05). From a total of 172 DEPs detected in cells exposed to the BEA standard (100 nM), 57 proteins displayed higher and 115 displayed lower levels of expression when compared to the control. In the presence of digested *Gentiana lutea* (DG), 51 proteins out of 145 showed higher expression, and in the combined exposure, 37 proteins were upregulated out of 139 with respect to the control. Among DEPs, 75 (28%) were solely expressed in the presence of BEA, 44 (16%) with the DG, and 30 (11%) with BEA + DG. On the contrary, a total of 71 (26%) features were common in all conditions (Figure 1). All DEPs obtained through statistical analysis are reported in the Supplementary Material.

### 2.2. Functional Analysis of Differentially Expressed Proteins

**Figure 2:**
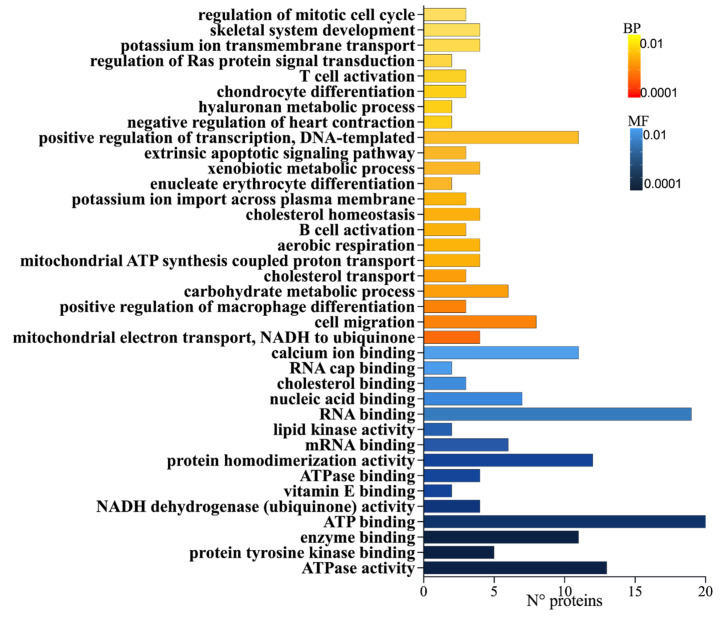
Gene ontology representation for biological processes (BPs) and molecular functions (MFs) of cells exposed to BEA related to the number of proteins and significance level; color gradient from the most significant *p* < 0.0001 (dark color) to the lowest *p* < 0.01 (light color).

Proteins expressed after exposure to BEA (100 nM), DG, and the combined exposure were identified in the analysis of biological processes (BP) and molecular functions (MFs) (Figure 2). The analysis reported the most significant BP for BEA exposure as the mitochondrial electron transport chain, in which NDUFV9, NDUFA9, NDUF6, and MT-ND2 were the most affected. Moreover, the regulation of transcriptional processes and cell migration displayed the biggest number of features implicated (*n* = 11 and *n* = 8, respectively). As regards MF, the most common was the ATPase activity involving 13 DEPs, whereas the highest number of proteins (*n* = 25) was observed in the ATP binding process, followed by RNA binding (*n* = 19). 

**Figure 3:**
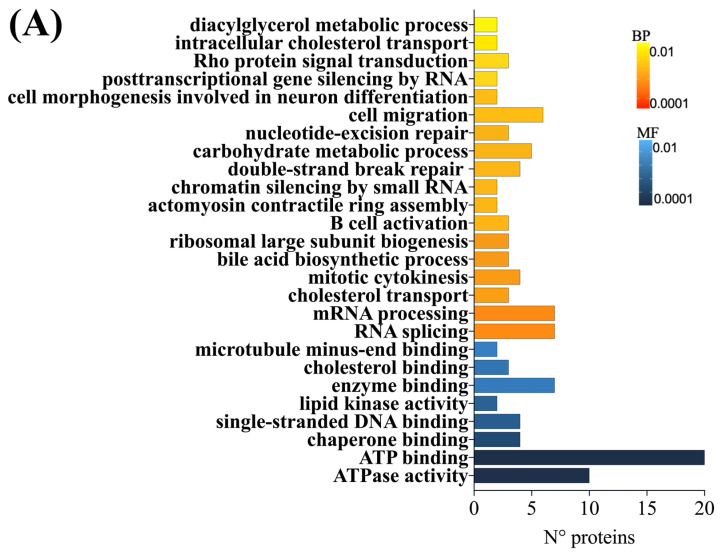

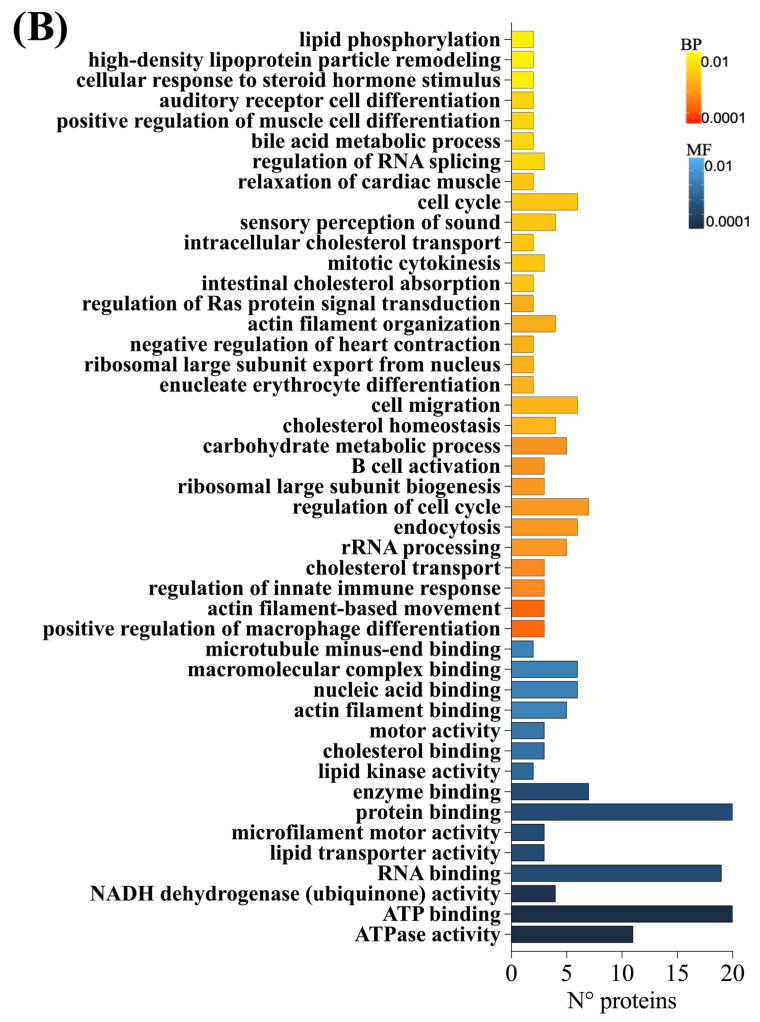
Gene ontology representation for biological processes (BPs) and molecular functions (MFs) of cells exposed to DG (**A**) and BEA + DG (**B**) related to the number of proteins and significance level; color gradient from the most significant *p* < 0.0001 (dark color) to the lowest *p* < 0.01 (light color).

When exposing cells to *G. lutea*, BP related to RNA splicing and mRNA processing were the most significant (*p* < 0.003) (Figure 3A). For both processes, the expression of AKAP17A, SON, EFTUD2, and FAM172A was lower with respect to the control (logFC < −4), whereas NRDE2, PQBP1, and SRRM1 were upregulated. As for molecular function, ATPase activity stands out once again, followed by ATP binding, reporting a total of 10 and 20 proteins altered, respectively. Likewise, in the third combined condition, positive regulation of macrophage differentiation was the most significant BP (*p* < 0.0048), with three proteins involved. Furthermore, seven proteins were found to be altered in the regulation of the cell cycle, including MDM2, RB1, SON, ID2 TRIOBP, MAPK12, PES1, and ZNF268 (Figure 3B). Also, in this case, MFs were equally observed, as in the second condition. Nevertheless, RNA binding covers the expression of 19 features. 

With respect to the main immune-related signaling pathways influenced, mitochondrial-associated pathways were the most significant and enriched in the presence of BEA. More specifically, complex I biogenesis, ion transport by P-type ATPases, respiratory electron transport, potassium channels, and ion channel transport stand out (Table 1). On the other hand, when cells were exposed to DG individually or in combination, the participation of RHO GTPases was found, with the highest number of features implicated (*n* = 10). 

**Figure 4:**
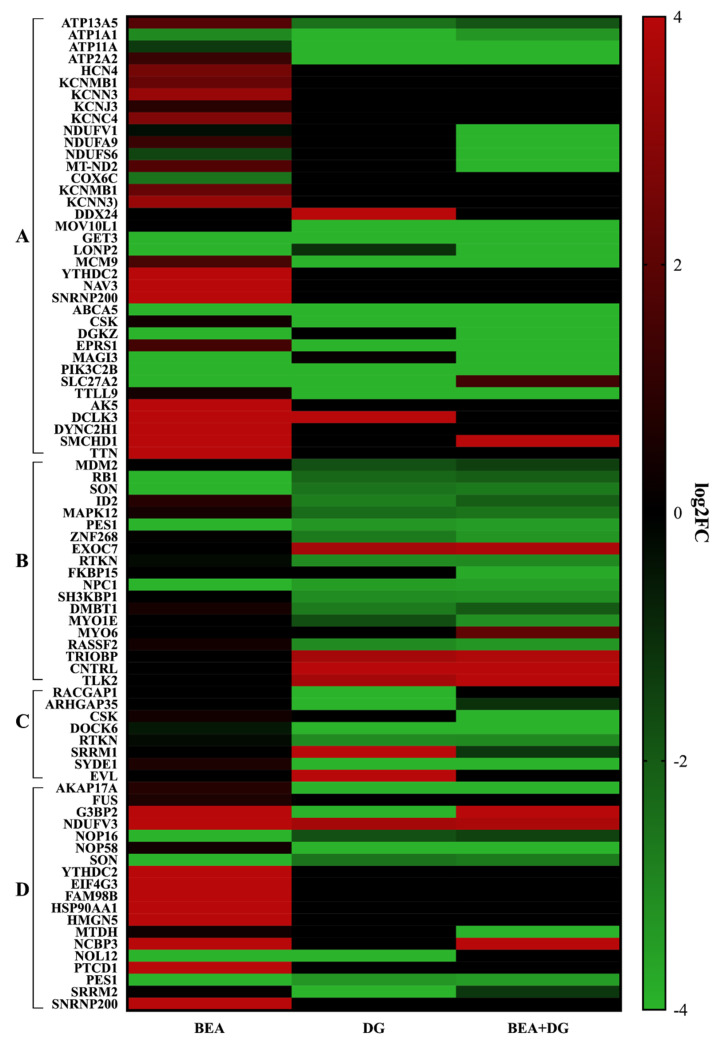
Heatmap representation of protein expression for mitochondrial and ATP-related activity (**A**), cellular processes (**B**), RHO GTPases cycle (**C**), and RNA binding (**D**) after BEA, DG, and BEA + DG exposure. The red-to-green gradient represents the logarithmic fold change value for upregulated (logFC = 4) and downregulated (logFC = −4) features.

After the identification of DEPs implicated in the main metabolic processes, a heatmap generated from proteomic data displayed downregulated (green) and upregulated ones (red) for Jurkat cells after BEA, DG, and BEA + DG exposure compared to matched controls (Figure 4). Proteins whose expression was strongly and significantly different in BEA belonged to mitochondrial electron transport chain complex I, complex IV, and ATP-related activity (Figure 4A). Similarly, most of the RNA-binding proteins (RBPs) were strongly upregulated after BEA exposure but downregulated when exposed to DG (Figure 4D). When exposing cells to the digested flower, RAS homolog (RHO) GTPases showed a significant alteration, as did the ones implicated in cellular processes in both conditions (Figure 4B,C).

## 3. Discussion

According to the EFSA’s latest scientific opinion on risks to human and animal health related to the presence of emerging mycotoxins in food and feed, chronic exposure to BEA might represent a potential concern for human and animal health [16]. Focusing on the alterations induced by this emerging mycotoxin on the immune system, it has been observed in studies in vivo to be concentrated in terms of the number and functional activity of T cells [17]. On the other hand, *Gentiana lutea* is a medicinal plant rich in several secondary metabolites that possess various therapeutic effects, such as anti-inflammatory, antioxidant, antimicrobial, and especially immunomodulatory activities [18,19]. In particular, studies in vivo have demonstrated that this plant at different concentrations could prime the immune system and support the immune response against chronic diseases [20,21]. Moreover, it has been shown that its bitter compound, amarogentin, reduces chronic inflammation progression by reducing the secretion of cytokines by T cells [22]. As for doses employed, concentrations of 0.01 mg of plant extract were linked to the inhibition of enzymes involved in immune system toxicity [23]. Herein, this research combines the assessment of BEA’s ability to modulate lymphoblastic T-cell proteomes after 7 days of exposure with an investigation of the beneficial contribution of *G. lutea* bioactive compounds to ameliorate this negative progression.

Starting with BEA, it is a secondary metabolite that is capable of integrating into diverse mammalian cells through the formation of complex structures with cations in the mitochondrial phospholipidic membrane [24]. In this sense, it has been previously demonstrated to induce an increase in intracellular Ca^2+^ concentration in leukemia cells, stimulating K^+^ channels and leading to cell shrinkage and death [25,26]. In particular, the inhibition of these channels, which provide the electrical force of divalent cations, negatively regulates T-cell activation [27]. Likewise, BEA altered the expression of genes coding for proteins of the electron transport chain (ETC) in vivo [28,29]. In this work, the expression of proteins with significant differences based on BP, MF, and related pathways after BEA exposure in Jurkat T-cells confirmed the modification of mitochondrial processes, such as the ETC and activated K^+^ channels (Figure 2, Table 1). As for ETC, four proteins were modified in abundance levels in complex I (NDUFV1, NDUFA9, NDUFS6, and MT-ND2), whereas only one belonged to complex IV (COX6C). Moreover, five features of K^+^ channels were upregulated up to three fold, including HCN4, KCNMB1, KCNN3, KCNJ3, and KCNC4. Remarkably, when adding *G. lutea*, the expression was not altered, but, on the contrary, in the combined exposure, a strong downregulation (LogFC = −4) of complex I proteins was observed (Figure 4A).

A fair number of significant proteins also belonged to the ATPase family (*n* = 11) and ATP binding (*n* = 24) in all conditions investigated (Figure 2 and Figure 3). ATPase proteins are a fundamental group of cellular catalyzers that transduce the chemical energy derived from ATP hydrolysis into transmembrane ionic electrochemical potential differences. This process, in turn, requires an ATP binding site that allows ATP molecules to interact and make enzymes catalytically active [30]. The activity of integral membrane transporter P-type ATPases, which maintain chemical gradients across membranes, was modified by the presence of the mycotoxin, highlighting the upregulation of ATP13A5 (LogFC = 1.82135) and ATP2A1 (LogFC = 1.24134). Likewise, members of the ATPase family, such as MCM9, YTHDC2, NAV3, and SNRNP200, increased their expression up to fourfold (Figure 4A). In both groups, in the presence of *G. lutea*, genes were downregulated. As for ATP binding, 8 proteins out of 13 (CSK, EPRS1, TTL9, AK5, DCLK, DYNC2H1, SMCHD1, and TTN) revealed a similar trend of being upregulated with BEA but not with DG (Figure 4A).

Focusing on both conditions, including BEA, another common MF in which a considerable number of features (*n* = 19) participated was RNA binding (Figure 2 and Figure 3). RBPs control all post-transcriptional regulatory mechanisms and are fundamental for the development, homeostasis, and functioning of the immune system [31]. A dysregulation in its activity entails the aberrant activation of immune responses, leading to the development of inflammatory and autoimmune diseases [32]. In this case, almost all entities were affected by BEA exposure when compared to the controls (Figure 4D), but, in contrast, such a condition was ameliorated once again by the addition of *G. lutea.*

DEPs related to cellular processes were modified in cell cycle regulation (*n* = 7), mitotic cytokinesis (*n* = 2), endocytosis (*n* = 6), and cell morphology (*n* = 4) in Jurkat cells exposed to *G. lutea* individually or in combination with BEA. Recently, healing properties linked to the biological activities of this plant were reviewed by Ponticelli et al. [33], indicating how its bioactive molecules attenuate cell activity and prevent several human illnesses. More specifically, its bitter compounds, such as amarogentin and gentiopicroside, were able to modulate cell cycle impairments at different stages and cellular homeostasis mechanisms in vitro and in vivo. In particular, a root extract of this plant blocked platelet-derived growth factor (PDGF-BB)-induced proliferation of rat aortic smooth muscle cells [34]. According to these findings, important oncoproteins involved in cell cycle regulation, such as murine double minute 2 (MDM2), mitogen-activated protein kinase 12 (MAPK12), zinc finger protein (ZNF268), and DNA-binding inhibitor (ID2), decreased in their expression down to −3.30-fold in both conditions (Figure 4B). On the contrary, they were slightly upregulated with BEA (log2FC > 0.0709), suggesting negative matching. In fact, it has been demonstrated that the overexpression of the above-mentioned enzymes is associated with the onset of several tumors [35,36,37,38]. 

Lastly, the results highlight the activity of the RHO GTPase protein cycle, and, in this case, with the DG (Table 1). These proteins are a major group of molecular activators that regulate several complex cellular processes by using a simple biochemical reaction. More specifically, they hydrolyze the GTP molecule to GDP by switching between the active state linked to GTP and the inactive one bound to GDP [39]. In the individual exposure, a total of 23 proteins were indicated in the RHO GTPase cycle, whereas a total of 11 were indicated in the combined one (Table 1). Among them, after DG exposure, standouts include the strong downregulation (logFC = −4) of GTPase activating protein 1 (RACGAP1) and Rho GTPase activating protein 35 (ARHGAP35), which are putative oncoproteins whose overexpression is linked with different tumor types [40,41]. Furthermore, dedicator of cytokinesis 6 (DOCK6) followed the same trend with *G. lutea* (logFC = −4) but not with the mycotoxin (logFC = −0.53). In fact, according to these findings, these proteins play a fundamental role in the immune surveillance mechanisms of human cells and act as guanine nucleotide exchange factors (GEFs) in various biological systems [42]. Likewise, the expression of C-terminal Src kinase (CSK) involved in modulation of immune function was strongly decreased in the combined exposure (logFC = −4) but not with BEA (logFC = 0.38) [43]. 

## 4. Conclusions

The exposure to BEA in vitro over 7 days showed a negative correlation with mitochondrial processes of complex I and IV, as well as ATP-linked activities and RNA binding, as previously described in in vivo models. On the contrary, *G. lutea* flower, in most cases, ameliorated these alterations induced by the mycotoxin. Moreover, its bioactive compounds attenuated cell cycle impairments through the downregulation of major oncoproteins such as MDM2, MAPK12, ZNF268, and ID2. Likewise, the RHO GTPase cycle stands out throughout the positive modification of immune surveillance mechanisms.

Based on the current results, *G. lutea* flowers could be a possible candidate for the realization of food enriched with natural extracts to mitigate mycotoxin activity. However, further investigations are needed to better explore the use of such functional food ingredients in vivo as active agents against BEA toxicity.

## 5. Material and Methods

### 5.1. Reagents

The reagent-grade chemicals and cell culture components used: RPMI-glutamax medium, penicillin/streptomycin, fetal bovine serum (FBS), and phosphate buffer saline (PBS) were purchased from Gibco by Life Technologies (Grand Island, NE, USA). BEA standard (mw: 783.95 g/mol, 97% purity) and methanol were obtained from Sigma–Aldrich (Madrid, Spain). Stock solutions of BEA (1 mg/mL) were prepared in methanol and maintained at −20 °C. The final concentration of BEA in the assay was achieved by drying the methanol using N_2_ flow at room temperature and diluting it in DMSO before introducing the mycotoxin into the culture medium. The final concentrations in the medium were 0.1% (*v*/*v*) for DMSO and 100 nM for BEA. 

For protein extraction and digestion, dl-dithiothreitol (DTT), ≥ 99.0%, Trizma^®^ hydrochloride (Tris-HCl) ≥99.0% purity), and trypsin, were acquired from Sigma Aldrich (St. Louis, MO, USA). Iodoacetamide (IAA), 98% was obtained from ACROS Organics™, Thermo Fisher Scientific (Waltham, NJ, USA). Thiourea (TU), 99% for lysis buffer preparation, was obtained from Thermo Fisher Scientific (Kandel, Germany), and urea (U), 99%, was acquired from FEROSA (Barcelona, Spain). For proteomics analysis, deionized Milli-Q H2O (<18 MU cm resistivity) was obtained from a Milli-QSP^®^ reagent water system (Millipore, Bedford, MA, USA). Acetonitrile (ACN) LC/ MS-grade OPTIMA^®^ (≥99.9% purity) was supplied by Fisher Chemical (Geel, Belgium). Formic acid (≥98%) was obtained from Sigma Aldrich (St. Louis, MO, USA).

### 5.2. Plant Material

*Gentiana lutea* flower samples were provided and harvested by “Parco Nazionale della Majella”, an Italian National Park in the Apennine area of the Abruzzo Region, in July 2021. These flowers present a characteristic yellow color, and the corolla is separated nearly to the base into five to seven narrow petals. The fresh flowers were freeze-dried by LyovaporTM L-200 (Buchi, Milan, Italy) at 0.200 mbar for 48 h and then ground using an IKA^®^ A11 basic analytical mill (IKA^®^-Werke GmbH & Co. KG, Staufen im Breisgau, Deutschland). The dry samples were stored at −20 °C until analysis.

### 5.3. In Vitro Digestion of G. lutea Flower

A static in vitro digestion system was carried out by employing a modified protocol from [44]. First, artificial saliva was prepared by mixing an inorganic solution containing 1 mL of KCl (89.6 g/L), 1 mL of KSCN (20 g/L), 1 mL of NaH_2_PO_4_ (8.8 g/L), 1 mL of Na_2_SO_4_ (57 g/L), 0.17 mL of NaCl (175.3 g/L), 2 mL NaHCO_3_ (84.7 g/L), and an organic solution consisting of 0.8 mL of urea (25 g/L), 29 mg of α-amylase, and 2.5 mg of mucin. The saliva solution’s pH was adjusted to 6.8 ± 0.2 and diluted to 50 mL with distilled water. Afterwards, 0.100 g of *Gentiana lutea* flower was mixed with 0.6 mL of saliva and 8.4 mL of Milli-Q water (Millipore, Bedford, MA, USA) in order to obtain a paste-like consistency, then introduced into an IUL Stomacher (IUL S.A, Barcelona, Spain) for 30 s to simulate the oral phase. Secondly, the gastric phase was initiated by acidifying the mixture to pH = 2 with a 6 N HCl solution and adding 54 µL of pepsin solution (1 g in 25 mL of 0.1 N HCl). Thereafter, samples were subjected to incubation under darkness for 2 h at 37 °C and slight agitation (100 rpm) with an orbital shaker (Infors AG CH-4103, Bottmingen, Switzerland). After the incubation time, the pH was adjusted to 6.5 ± 0.2 using 1 N NaHCO_3_, and 125 µL of bile salts/pancreatin mix (0.1 g of pancreatin, 0.625 g of bile salts, and 25 mL of 0.1 N NaHCO_3_) were added. The samples were incubated again under the same conditions and subsequently centrifuged (4500 rpm for 10 min at 4 °C). The supernatant was collected to obtain the intestinally digested flower.

### 5.4. Cell Culture and Exposure Conditions

Cell culture was performed as previously described [45]. Briefly, Jurkat cells (ATCC-TIB152) were maintained in RPMI-glutamax medium supplemented with 100 U/mL penicillin, 100 mg/mL streptomycin, and 10% (*v*/*v*) inactivated FBS. Incubation conditions were pH 7.4 and 37 °C under 5% CO_2_ and a 95% air atmosphere at constant humidity. The culture medium was changed every 2 days. The absence of mycoplasma was checked routinely using a Mycoplasma Stain Kit (Sigma Aldrich, St Louis, MO, USA). Before contamination, cells were grown independently to an approximate density of 2 × 10^7^ cells/flask. In cell culture, 3 biological replicates of each condition (DMSO 0.1%, BEA 100 nM, (DG) 2%, and BEA + DG 2%) were performed. A total of 2 ×10^5^ cells in 3 mL per well were seeded in 12-well plates using the corresponding conditions (Table 2), with DMSO 0.1% exposure as a control. Cells were treated for 7 days, and the medium was changed every 2 days, as previously described [46]. Once the exposure time had elapsed, the culture medium contained in each well of the plate was collected, added to a 15 mL conical centrifuge tube, and centrifuged at 1200 rpm for 4 min. Then, the supernatant was discarded.

### 5.5. Protein Extraction and Sample Preparation

Each biological replicate pellet was diluted using 100 μL of lysis buffer (8 M urea/2 M thiourea/50 mM tris-HCl). The procedure continued as explained in Cimbalo et al. [46]. To sum up, sonication was carried out on ice for 3 to 5 times in a methanol:H_2_O mixture (USC 1200D ultrasonicator, VWR, International bvba, Leuven, Belgium). Later, sonicated samples were centrifuged at 4 °C and 13,000 rpm for 30 min. Biological replicates were transferred to sterile LoBind tubes for DNA, RNA, and proteins in order to decrease the concentration of plastic particles and sample attachment. Protein concentration in the supernatant was measured at a wavelength (λ) of 280 nm with a NeoDot UV/VIS Nano Spectrophotometer by NeoBiotech (Nanterre, France; Quimigen, Madrid, Spain). The purity ratio was considered A260/A280, specific for proteins, to check that there were no other cellular components, such as RNA or DNA. Finally, the samples were diluted with the lysis buffer (8 M urea/2 M thiourea/50 mM tris-HCl) to obtain a protein concentration of 1 mg/mL.

To break protein disulfide bonds, DTT 200 mM was used at 5 μL/sample, followed by incubation for 1 h at 60 °C in an Eppendorf ThermoMixer C (Merck, Darmstadt, Germany). Afterwards, a 20 μL/sample of IAA 200 mM was added, and they were incubated for 30 min at 37 °C in order to break the thiol groups of the proteins. Finally, trypsin was added at a concentration of 1 mg/mL, and the digestion was carried out overnight at 37 °C (16–18 h). Digestion was stopped using 5% acetic acid, and samples were frozen at −80 °C for 3–4 h. The last step was the lyophilization of the samples for 2 h at −40 °C under vacuum pressure of 0.080 mbar in a lyophilizer Freezone 2.5 benchtop freeze dryer (Labconco, Kansas City, MO, USA). The lyophilizate was resuspended in 100 μL of H_2_O: ACN in a ratio of 98:2 % *v*/*v* to finally obtain a concentration of 100 μg/mL.

### 5.6. Q-TOF Mass Spectrometry and Data Analysis

Two technical replicates were prepared in vials for each biological sample (100 μg/mL) and injected into a liquid chromatography instrument (Agilent 1200 Infinity Series LC system) coupled to a quadrupole time-of-flight accurate mass (Q-TOF) (Agilent 6540 UHD) equipped with a Dual Jet Stream electrospray ionization (Dual AJS ESI) (Agilent Technologies, Santa Clara, CA, USA). Peptide separation was performed using a C18 bioZenTM column (Phenomenex) at 2.6 μm, 120 Å, and 50 × 2.1 mm. Injection volume, total run, phases, chromatographic gradient, and QTOF-MS parameters are explained in [47].

Subsequently, proteomics data processing was carried out from the obtained spectra using the Spectrum Mill MS Proteomics Workbench software package BI.07.09 (Agilent). The SwissProt human database was employed for MS/MS spectra research and validation using 1.2% FDR as the criteria. Validation parameters used started with trypsin specificity, maximum missed cleavages 2, fixed modification carbamidomethylation (C), variable modification oxidized methionine (M), minimum matched peak intensity 50%, precursor mass tolerance ±20 ppm, product mass tolerance ±50 ppm, maximum ambiguous precursor charge 3, minimum detected peaks 3, and precursor isolation purity >70%. Peptide identification was based on a maximum FDR of 1.2% for each LC run, a minimum peptide length of six amino acids, and a precursor charge range of 2–6. These data were also searched with ±20 ppm precursor and ±50 ppm fragment ion tolerance. 

Afterwards, identified features were statistically filtered by using Mass Profiler Professional 15.0 version software (Agilent, 2021) in order to determine differences in abundance between the conditions and the control. Contrasts for each condition between the experimental mycotoxin dose and the control were analyzed using the unpaired t-test with Benjamin-Hochberg adjustment. Results were considered significant with fold change (FC) values ≥ 0.7 for upregulated proteins and FC ≤ 0.7 for downregulated ones; *p*-value cut-offs were <0.05.

Lastly, each DEP was searched in the UNIPROT database by its identification code mapped for *Homo sapiens*. With the given code, gene ontology (biological processes and molecular functions) analysis was conducted using the DAVID database [48,49]. The analysis of metabolic pathways was performed using the Reactome pathways tool integrated in the DAVID database. Graphical representations of the data were created with GraphPad Prism software version 8.0.0 (San Diego, CA, USA). A Venn diagram was generated using the Venny 2.1 interactive tool [50].

## Figures and Tables

**Table 1:** Reactome pathway results using DEPs in Jurkat cell proteomics analysis after exposure to BEA, DG, and BEA + DG by entity number, count, (%), *p*-value, and fold enrichment.

**Term**	**Count**	**%**	***p*-Value**	**Fold Enrichment**
BEA				
Complex I biogenesis	4	2.4	1.8 × 10^−2^	7.1
Ion transport by P-type ATPases	4	2.4	1.8 × 10^−2^	7.1
Respiratory electron transport	5	3	1.8 × 10^−2^	4.9
Potassium Channels	5	3	1.8 × 10^−2^	4.9
Ion channel transport	6	3.6	2.7 × 10^−2^	3.3
Respiratory electron transport, ATP synthesis by chemiosmotic coupling, and heat production by uncoupling proteins.	5	3	3.6 × 10^−2^	4
NOD1/2 Signaling Pathway	3	1.8	3.6 × 10^−2^	8.2
Ca^2+^ activated K^+^ channels	2	1.2	5.0 × 10^−2^	22.5
The citric acid (TCA) cycle and respiratory electron transport	5	3	5.1 × 10^−2^	2.8
DG				
RHOD GTPase cycle	4	2.7	1.0 × 10^−2^	8.9
Synthesis of bile acids and bile salts via 7alpha-hydroxycholesterol	3	2.1	1.7 × 10^−2^	15
RHO GTPase cycle	9	6.2	3.2 × 10^−2^	2.4
Synthesis of bile acids and bile salts	3	2.1	2.8 × 10^−2^	11.2
TLR3-mediated TICAM1-dependent programmed cell death	2	1.4	4.6 × 10^−2^	42.4
Bile acid and bile salt metabolism	3	2.1	4.8 × 10^−2^	8.5
Ion transport by P-type ATPases	3	2.1	7.2 × 10^−2^	6.7
TRIF-mediated programmed cell death	2	1.4	7.5 × 10^−2^	25.5
Peroxisomal protein import	3	2.1	8.6 × 10^−2^	6.1
Cell-cell junction organization	3	2.1	8.8 × 10^−2^	6
Signaling by Rho GTPases	10	6.9	9.7 × 10^−2^	1.8
BEA + DG				
Ion transport by P-type ATPases	4	2.9	9.9 × 10^−3^	8.9
Sensory processing of sound by inner hair cells of the cochlea	4	2.9	1.7 × 10^−2^	7.3
Sensory processing of sound	4	2.9	2.1 × 10^−2^	6.7
Ion channel transport	5	3.6	5.7 × 10^−2^	3.4
RHO GTPase cycle	8	5.8	6.1 × 10^−2^	2.3
RHOD GTPase cycle	3	2.2	6.6 × 10^−2^	7
rRNA modification in the nucleus and cytosol	3	2.2	8.2 × 10^−2^	6.2
Peroxisomal protein import	3	2.2	8.7 × 10^−2^	6

**Table 2:** Exposure conditions of Jurkat cells to DMSO, BEA (nM), digested *Gentiana lutea* (%), and combined exposures.

Conditions	BEA (nM)	DG (%)	DMSO (%)
DMSO 0.1%	-	-	0.1
BEA in DMSO 0.1%	100	-	0.1
DG 2%	-	2	0.1
BEA in DMSO 0.1% + DG 2%	100	2	0.1

## Supplementary Materials

The following supporting information can be downloaded at: https://www.mdpi.com/article/10.3390/toxins15090538/s1.
